# A Cease in Shift Work Reverses Arterial Stiffness but Increases Weight and Glycosylated Hemoglobin A 5-Month Follow-Up in Industry

**DOI:** 10.3390/jcdd9060190

**Published:** 2022-06-12

**Authors:** Marit Skogstad, Hans Christian D. Aass, Lars-Kristian Lunde, Øivind Skare, Per Anton Sirnes, Dagfinn Matre

**Affiliations:** 1 National Institute of Occupational Health (STAMI), Box 5330 Majorstuen, 0304 Oslo, Norway; lars-kristian.lunde@stami.no (L.-K.L.); oivind.skare@stami.no (Ø.S.); dagfinn.matre@stami.no (D.M.); 2 The Blood Cell Research Group, Department of Medical Biochemistry, Oslo University Hospital, 0450 Oslo, Norway; h.c.aass@medisin.uio.no; 3 Ostlandske Hjertesenter, 1523 Moss, Norway; pas@cardio.no

**Keywords:** shift work, cardiovascular, arterial stiffening, occupational health

## Abstract

Background: Literature suggests an association between shift work and cardiovascular disease (CVD). Limited evidence is available on how a cessation of shift work affects CVD risk factors. Aim: We investigated whether a five-month plant shutdown affected CVD risk factors in 30 industrial shift workers. Methods: We collected demographic data, self-reported data on physical activity (PA) and medical history by questionnaire. Pre- and post-plant shutdown, we measured blood pressure (BP), heart rate, lipids, glycosylated hemoglobin (HbA1c) and C-reactive protein (CRP). Additionally, we collected markers of inflammation, Matrix metalloproteinase-9 (MMP-9), Interleukin-6 (IL-6), Monocyte chemoattractant protein-1 (MCP-1), Tumor necrosis factor-alpha (TNF-α), P-selectin, Interleukin-1 beta (IL-1β), and Interleukin-23 (IL-23). We also examined arterial stiffness (central blood pressure, augmentation pressure, and pulse wave velocity) by means of SphygmoCor^®^ (AtCor Medical Pty Ltd., Sydney, Australia). We monitored sleep by actigraphy prior to and after plant shutdown, with additional registration of sleep quality and assessment of insomnia symptoms. Results: After five months of plant shutdown, we found that HbA1c increased by 1.9 mmol/mol, weight by 1 kg and MCP-1 by 27.3 pg/mL, all unexpectedly. The other markers of inflammation did not change during shutdown, but CRP decreased close to significant levels. There were no changes in lipids during follow-up. Pulse-wave velocity (PWV) was reduced from 8.1 m/s (SD = 1.5) to 7.6 m/s (SD = 1.5), *p* = 0.03. The workers reported fewer signs of insomnia after shutdown. Conclusions: Our findings suggest that a five-month cessation in shift work increases weight and HbA1c, but also improves insomnia symptoms and reverses arterial stiffening.

## 1. Introduction

Increasing globalization has resulted in the increased frequency of shift work with night shifts and long working days [1]. A common definition of shift work is work that includes working hours beyond conventional daytime hours. With regard to night work, several definitions are suggested. One definition suggests that such work comprises more than seven consecutive hours including midnight and five o’clock in the morning. Another suggests that night work should include more than three hours, encompassing eleven at night and three o’clock in the morning [2]. Further, according to the Norwegian Working Environment Act, night work is between nine o’clock in the evening and six o’clock in the morning.

Rotating shift work refers to work in which the worker rotates between day, and afternoon/evening shifts, including or not including night shift [1].

Shift work increases the risk of workplace accidents, type 2 diabetes, weight gain, and some cancers [3,4]. A recent systematic review with meta-analyses discloses an association between shift work and CVD, suggesting a possible increase in risk with increasing number of years in shift work [5].

Disrupted circadian rhythm and sleep deficit, known to affect shift workers, could result in metabolic and endocrine changes. Such changes may lead to increased sympathetic activity, activation of the hypothalamic–pituitary axis, increased inflammation, disturbed cortisol, glucose metabolism, and changes in the release of ghrelin and leptin, thus affecting appetite regulation. These alterations affect the metabolic milieu of shift workers and may result in hypertension and other components of the metabolic syndrome, such as overweight/obesity and type 2 diabetes [4].

We have previously demonstrated that increasing number of years with shift work in the insulation industry is associated with increased maximal intima media thickness (max IMT) and elevated C-reactive protein (CRP) [6]. The aim of the present study was to determine the short-term consequences between shift work, insomnia symptoms and CVD risk factors. 

Our primary hypothesis is that a cessation of shift work reverses the risk of early manifestations of CVD and thus has a positive impact on cardiovascular health. Our secondary hypothesis is that a cessation of shift work improves insomnia symptoms. 

## 2. Materials and Methods 

Study design and population. This study was designed as part of a larger study with full description of recruitment and measurement methods provided elsewhere [7]. Briefly, we recruited participants from two insulation material plants (A + B) in Eastern Norway [7]. There were all together 50 eligible shift workers at plant B, a plant that planned a shutdown due to technical reasons, and 30 of these shift workers agreed to participate in the present study, Table 1. According to the protocol [7], we excluded persons with serious medical conditions. Prior to shutdown ultimo June 2020, we examined the participants during day- and evening shifts or when they were off duty. We performed all tests at the plant. Due to the COVID-19 pandemic, we performed all post-tests during day-time (before the re-opening of the plant in primo December 2020) at the Occupational Health Services in the city where the plant was situated. 

Medical history. We collected background data and medical history by questionnaire. We measured weight in kg using a Seca 22089 balance (Hamburg, Germany). We assessed physical activity (PA) by asking the participants to report, in minutes per week, the total amount they were engaged in PA with a low/moderate intensity (walking) and a high degree of intensity (running, bicycling). Total tobacco consumption for each participant, by means of pack-years, was calculated: In which one pack of 20 cigarettes daily consumed for one year equals one pack-year.

Shift work exposure. The shift workers follow a schedule lasting for five weeks in which day, evening and night shifts rotate in a clockwise manner. The ordinary shift plan comprises seven day shifts and seven night shifts, of which four are consecutive 8-h shifts and three, are consecutive 12-h shifts. The ordinary plan also includes four evening shifts. Seven months prior to shutdown the shift schedule included only night (five shifts in a 5-week period) and day shifts of 12 h duration. This plan changed seven weeks prior to shutdown. Then, the shift workers were divided in two; one group consisting of 20 workers worked 10 night shifts of 8 h duration for a period of five weeks, while the other group of workers worked six night shifts of 12 h duration during the same period. During the 5-month plant shutdown, the workers no longer worked shifts. 

Brachial blood pressure (BP) and resting heart rate (RHR). On both occasions, while the subject was sitting, we measured blood pressure (BP) and resting heart rate (RHR), after five minutes of rest, on the left arm three times in one-minute intervals. We used the mean of three measurements of the systolic (sBP) and the diastolic pressure (dBP) in the statistical analysis. We measured blood pressure and RHR by means of BpTRU^®^ (Bp TRU medical devices, Coquitlam, BC, Canada).

Arterial stiffness. We assessed central blood pressure on both occasions, augmentation pressure (AP), pulse pressure (PP) and pulse-wave velocity (PWV) by SphygmoCor XCEL^®^ (AtCor Medical Pty Ltd., Sydney, Australia). We performed the measurements according to the manufacturer’s recommendations (www.atcormedical.com, accessed on 30 April 2012) and used the mean of three measurements in the analysis. 

Blood analyses. Both in June and December 2020, we analyzed glycosylated hemoglobin (HbA1c) and lipids (cholesterol, low-density lipoprotein (LDL), high-density lipoprotein (HDL)) and CRP, by using ethylene diamine tetra acetic acid (EDTA) blood and whole blood (gel tubes). We centrifuged the gel tubes at 1240× *g* (3000 r/min) for 10 min within 60 min after venous blood collection. The Department of Medical Biochemistry Oslo University Hospital analyzed the samples within 48 h. To assess HbA1c (EDTA blood), the laboratory utilized a Tosoh G7 HPLC analyzer (Tosoh Bioscience, Inc. San Francisco, CA, USA). The analytical variation is 1.7%. Furthermore, we analyzed Cholesterol, LDL and HDL in serum by enzymatic colorimetric method in the Cobas 8000. Analytical variation coefficients are 3.0%, 4.0% and 3.5%, respectively. We assessed CRP in serum by particle-enhanced immune turbidimetric method on Cobas 8000 (Cobas 8000 Modular Analyzer Roche Diagnostics, (www.roche.com, accessed on 25 October 2011). The analytical variation for CRP is 8.0%. 

Measurement of plasma cytokine levels. A one plex MMP-9 kit (cat. no.: hrQABfLV, R&D system, Abingdon, UK) and a custom-made six plex kit (cat. no.: EqllRJyV, R&D system, Abingdon, UK) that included MCP-1; IL-6, P-Selectin, IL-1β, IL-23 and TNF-α, were used to screen all samples from the study participants. All samples, both those from June 2020 and of December 2020 were thawed, left on ice, and spun down at 10,000× *g*/10 min/4 °C. The supernatant was further diluted 1:50 (MMP-9) and 1:1 (6 plex) and 50 microliters of diluted sample were loaded onto plate. We analyzed all samples in duplicate and the two sets of samples from all participants were included on the same plate. The target protein levels were determined using a Luminex IS200 instrument (Bio-Rad, Hercules, CA, USA). To ensure reliable low-level cytokine detection, two additional standard points were included to the lower end of standard curve. We performed all washing steps with an automated magnetic plate washer (Bio-plex Pro wash station, Bio-Rad Laboratories, Inc., Hercules, CA, USA). Intra- and inter-percent (%) coefficient of variation (CV) was calculated based on an in-house spiked control and ranged from 0.9 to 6.1 (intra %CV) and from 2.3 to 11.3 (inter %CV).

Sleep measurements. We monitored sleep objectively via wrist-worn actigraphy (AX3, Axivity Ltd., Newcastle upon Tyne, UK) and subjectively by a smartphone-based sleep diary derived from the Consensus Sleep Diary-Core [8]. Each evening at 9pm, the participants received an SMS. Clicking on a URL provided in the message opened a web browser on the smartphone. The sleep diary was collected for three weeks before plant shutdown and for two weeks after re-opening. It included questions about time trying to sleep (“lights off”), sleep latency, time of final awakening (“lights on”), number of awakenings (NA), and time awake after sleep onset (WASO). Total sleep time (TST) was calculated objectively based on actigraphy [9]^,^ and subjectively based on the sleep diary by subtracting sleep onset latency and wakefulness after sleep onset from the difference between the time trying to sleep and the time of final awakening [10]. Furthermore, insomnia disorder symptoms were measured with the Bergen Insomnia Scale (BIS) [11]. This is a questionnaire with six items assessing difficulty to initiate and maintain sleep, at both occasions. Here, we calculated a sum score by adding the score for each question in the questionnaire. Higher total sum indicates more insomnia symptoms.

Statistical analysis. To determine whether the cessation of shift work was associated with changes in CVD measurements or sleep measurements, we chose a paired comparison approach. For CVD measurements, we applied Student’s paired *t*-test to assess differences before versus after closure of plant. For sleep measurements, we applied a mixed model approach, taking advantage of the day-to-day structure of the actigraphy and diary data. Time (before versus after closure) was entered as a fixed factor in the model. Residuals were tested for normality and transformed if relevant. We conducted all analyses using SPSS v. 25 (IBM SPSS, Armonk, NY, USA) and Stata (v. 16.1, College Station, TX: StataCorp USA). To correct for multiple comparisons of cardiovascular outcome measures, the significance level (expressed as *p* value) was corrected using a false-discovery rate procedure [12]. 

Since disturbed sleep represents a potential mechanism between shift work and CVD risk factors, a post hoc sensitivity analysis was performed. For each CVD and sleep variable exhibiting a significant change from before to after shutdown, new variables were calculated as delta measures (post minus pre). The association between CVD and sleep variables were then analyzed with bivariate non-parametric correlation analyses. 

## 3. Results 

**Figure 1:**
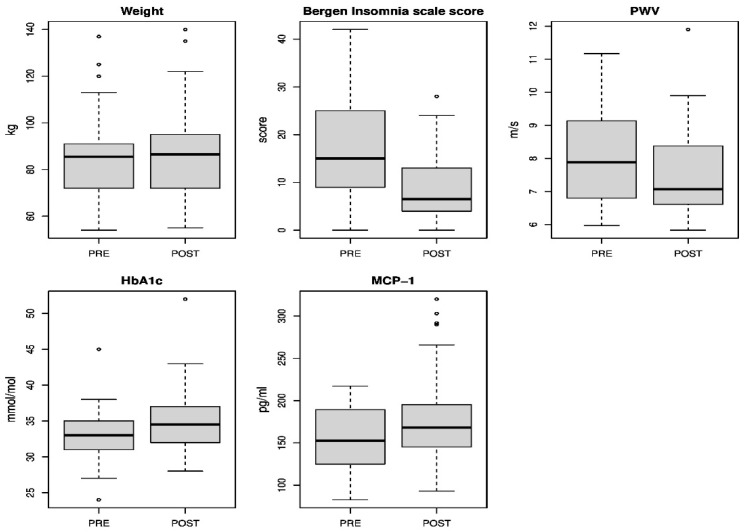
Boxplot of the change in selected outcome variables (Weight, BIS-scale, PWV, HbA1c and MCP-1) during the 5-month cessation of shift work. Results are reported as median, the interquartile range (the distance between the upper and lower quartiles) and possible outliers.

Demographic characteristics of the study population are shown in Table 1, Figure 1.

Brachial blood pressure and resting heart rate. Table 2 shows blood pressure (sitting and supine) on both occasions. No difference was found from before to after shutdown. 

Arterial stiffness. Augmentation pressure did not change during follow-up. PWV decreased significantly from 8.1 m/s before to 7.6 m/s after shutdown (mean diff: −0.45, 95% CI: −0.15, −0.75, Table 2, Figure 1). 

Blood analysis. We found no differences comparing lipids before and after shutdown. HbA1c increased significantly and CRP tended to decrease, Table 2, Figure 1. 

Markers of inflammation. MCP-1 increased during shutdown. As for the other markers of inflammation, MMP-9, P-selectin, TNF-α, IL-1β, IL 23 and IL 6, we found no significant change in plasma levels during shutdown, Table 2, Figure 1.

Sleep measurements. The workers reported significantly fewer insomnia symptoms after plant shutdown, as compared to before plant shutdown (Bergen insomnia scale), Table 2. In their diaries, the workers tended to report fewer awakenings, less time awake and longer sleep duration after plant shutdown, versus before plant shutdown, Table 2. Actigraph measurements of awakenings (NA), time awake (WASO) and sleep duration (TST) did not differ between before and after plant shutdown, Table 2, Figure 1.

Sensitivity analysis. Spearman correlation analyses were run between sleep (Bergen insomnia scale score) and CVD (PWV, HbA1c, MCP-1) measurements. A significant negative association was found between insomnia score and MCP-1 (rho = −0.54, *p* = 0.0026). No associations were found between insomnia score and PWV or HbA1c (*p* > 0.8). 

## 4. Discussion 

A five-month cessation in exposure to shift work in autumn 2020 in industry improves sleep quality and reduces arterial stiffness. However, being out of work increases HbA1c, weight and MCP-1, but the other markers of inflammation remain unchanged. We did not find any changes in lipids during follow-up. 

We have previously found an association between many years of shift work and increased intima media thickness [6]. Increased intima media thickness represents intima changes in the arterial vessels, reflecting atherosclerosis characterized by a progressive narrowing of the arteries’ lumina. Atherosclerosis is closely associated with arteriosclerosis, which appears when the arterial walls stiffen and thicken with age due to loss of elastic fibers and increased fibrosis in the media of the arterial vessel. Arterial stiffness is one of the earliest manifestations of vascular damage [13]. In the present study, we measured arterial stiffness by means of pulse-wave velocity (PWV). During the five-month cessation of shift work there was a reduction in PWV irrespective of increases in weight and BP along with less physical activity (PA), all known to be associated with the opposite, namely an increase in PWV [13]. The decrease in PWV during plant shutdown could be due to a reversible change in tonus within the intima or less inflammation. Initially at baseline in 2018, we found that the number of years as a shift worker was associated with increased levels of CRP [6]. A biological plausible reduction of CRP during the five-month follow-up could be due to better sleep quality since the workers reported fewer symptoms of insomnia during plant shutdown. Short sleep duration is associated with higher levels of inflammatory markers [14], and an association between inflammation and arterial stiffness has been described [15]. A possible explanation for the decrease in arterial stiffness is less inflammation related to better sleep quality and an increase in sleep duration (albeit not statistically significant). 

A major finding during the five-month shutdown of the plant was an increase in HbA1c, weight and the diabetogenic MCP-1. Circadian misalignment among night shift workers could result in altered timing of meals and affects the hormones leptin and ghrelin. These hormones are involved in regulation of food intake and appetite stimulation. An alteration of these hormones promotes weight gain [16]. Although the shift workers in the present study had a five-month break in night shift work, they might have remained in a vicious habitual circle regarding circadian rhythm and food habits. This could explain the weight gain among the workers, also described in shift workers who are followed prospectively [17]. Increases in HbA1c are linked to type 2 diabetes, described to be prevalent among shift workers [4], and a continual disturbance of the circadian rhythm or even bad habits cannot be ruled out in this group of workers. Furthermore, physical activity (PA), which tended to decline during the follow-up period, is related to insulin sensitivity [18]. It is important to note that the five-month cessation of shift work coincided with the COVID-19 pandemic. During the pandemic, people in Norway were allowed to move outdoors without any restrictions, but for many months, other parts of society closed, and along with this, fitness centers, possibly resulting in PA reduction, increase in sedentary behavior and disturbed sleep patterns on a general basis [19]. Thus, during plant shutdown, the workers may have spent a lot of time at home suggesting that the present results not only reflect a cessation of shift work but also the complete cessation of work.

A strength of the present paper is the study of early manifestations of CVD, examining many parameters and using advanced modern methodology. One cannot rule out self-selection of fit, highly-motivated shift workers wanting to take part in the study since only 60% of the shift workers agreed to participate. However, the workers in this study did not differ from the other group of shift workers at plant A, where more than 80% of the total number of workers attended [5]. Another strength of the study is its prospective design with a five-month cessation of the exposure factor, shift work. 

A limitation of the study is that PA was self-reported. Further, we did not measure covariates related to CVD, such as alcohol intake and eating habits. This presents a limitation since the literature suggests the occurrence of altered eating habits among shift workers [3]. We cannot rule out bias of outcome variables due to diurnal variation, but we mostly examined the workers between 10 am and 6 pm during the first round and between 9 am and 4 pm at follow-up. Thus, there is an overlap of registration times which would reduce the possibility of systematic errors. Another limitation of the study is that the plant shutdown was not associated with actigraphy-measured number of awakenings or time awake. The differences between self-reported and registered sleep quality may be attributed to problems in the algorithms calculating these measures in the actigraphs. The lack of a control group who were not exposed to plant shutdown represents another limitation. However, since the workers were followed for five months during the cessation of “exposure”, namely shift work, they serve as their own controls reducing the inter individual variability. Thus, we consider the design acceptable. A possible bias of the study affecting the workers’ psychological wellbeing could be an economic burden placed on the workers due to lost income following work cessation. The workers were guaranteed an income similar to their pre-work cessation income, thus we believe the economic concerns of the participants during follow-up were alleviated.

## 5. Conclusions 

A five-month cessation of shift work increases body weight along with HbA1c levels and reverses arterial stiffness. Decreasing the number and length of night shifts and the total number of years in such work may imply a reduced risk for cardiovascular disease in these workers. 

## Figures and Tables

**Table 1:** Baseline characteristics among shift workers (N = 30) participating in the study.

Variables	Shift Workers					
	Number	Mean	Median	Min	Max	SD
Age (years)		41.3	40	24	64	12.3
Women	1					
Weight		86.7	85.5	54	137	19.2
BMI (kg/m^2^)		26.3	26.2	18.7	35.3	4.3
Pack-years		6.0	1.12	0	37	9.8
Daily smokers	5					
Number of years as shift worker		15.9	14.5	2	35	9.8
Physical activity (PA), high intensity (min/week)		101	90	0	600	125

**Table 2:** Cardiovascular and sleep outcomes among shift workers (N = 30), pre- and post-shutdown values. The last six sleep variables were measured at multiple occasions post and pre: Subjective NA, n, (N pre = 203/post = 146), Objective NA, n, (N pre = 137/post = 103), Subjective WASO, min (N pre = 201/post = 148), Objective WASO, min (N pre = 137/post = 102), Subjective TST, hrs (N pre = 201/post = 146), Objective TST, h (N pre = 137/post = 103).

Variable	Pre-Shutdown		Post-Shut Down				Change (Post Minus Pre) during Five Months		
	Mean	SD	Mean	SD	*p*-Value	*p*-Value ^†^	Mean	SD	95% CI
**CVD**									
Weight	86.7	19.2	87.7	20.1	0.034 *		1.0	2.5	**0.085, 1.98**
BMI	26.3	4.3	26.6	4.5	0.03 *		0.3	0.7	**0.04, 0.79**
PA, high intensity (min/week)	100	125	85	116	0.55	N.C.	−15.8	143	−69.2, 37.6
Systolic BP (mmHg)	123.2	14.0	127.3	15.2	0.058	0.32	4.1	11.4	−0.14, 8.35
Diastolic BP (mmHg)	81.4	10.0	83.5	8.9	0.11	0.38	2.1	6.8	−0.46, 4.59
Supine systolic BP (mmHg)	125.5	12.1	126.2	13.1	0.74	0.94	0.7	12.0	−3.76, 5.24
Supine diastolic BP (mmHg)	74.8	9.3	74.3	8.3	0.58	0.96	−0.56	5.48	−2.61, 1.49
Systolic aorta pressure (mmHg)	112.7	12.0	112.7	11.9	0.97	0.97	0.06	9.4	−3.45, 3.57
Diastolic aorta pressure (mmHg)	75.3	9.6	75.2	8.4	0.94	0.97	−0.09	6.0	−2.32, 2.15
Supine RHR (bpm)	63.7	8.5	65.4	10.1	0.30	0.60	1.77	9.2	−1.67, 5.21
Augmentation pressure (mmHg)	8.4	6.3	8.0	5.0	0.65	0.94	−0.44	5.1	−2.35, 1.48
Pulse pressure (mmHg)	37.2	6.5	37.5	7.4	0.79	0.97	0.34	7.1	−2.29, 2.98
Pulse-wave velocity (m/s) ^a^	8.1	1.5	7.6	1.5	0.005	0.03 *	−0.45	**0.8**	**−0.75, −0.15**
CRP (mg/L)	2.6	2.9	1.8	2.2	0.09	0.38	−0.72	2.2	−1.55, 0.12
Cholesterol (mmol/L)	4.8	1.0	4.7	0.8	0.67	0.94	−0.07	0.9	−0.40, 0.25
HDL (mmol/L)	1.2	0.3	1.2	0.3	0.25	0.54	−0.03	0.1	−0.09, 0.23
LDL (mmol/L)	3.1	0.9	3.0	0.7	0.68	0.94	−0.06	0.8	−0.35, 0.23
HbA1c (mmol/mol)	32.9	4.2	34.8	4.9	<0.001	<0.001 **	1.93	1.7	**1.29, 2.58**
MMP-9 (pg/mL) ^b^	64,233	34,834	73,592	37,216	0.12	0.38	9359	31,569	−2649, 21,368
P-selectin (pg/mL) ^b^	26,260	7716	26,359	6413	0.87	0.97	48.7	3110	−1084, 1282
TNF-α (pg/mL) ^b^	12.8	2.7	13.1	3.1	0.22	0.54	0.31	1.3	−0.20, 0.82
IL-1β (pg/mL) ^b^	19.1	4.1	19.2	4.0	0.95	0.97	0.03	2.4	−0.87, 0.93
IL-23 (pg/mL) ^b^	204	109	194	110	0.25	0.54	−9.72	44.1	−26.5, 7.0
IL-6 (pg/mL) ^b^	5.6	1.5	5.5	1.4	0.64	0.94	−0.08	0.9	−0.43, 0.27
MCP-1(pg/mL) ^b^	157	38.4	185	59.9	0.002	0.03 *	27.3	44.1	**10.5, 44.0**
**Sleep**									
Bergen Insomnia Scale Score, (0-42)	16.5	10.3	8.9	6.8	<0.001 **	N.C.	−7.5	10.5	**−11.44, −3.63**
Subjective NA, n	1.4	1.4	1.1	1.4	0.06		−0.25		−0.50, 0.01
Objective NA, n	11.3	4.8	11.1	4.8	0.81		−0.15		−1.36, 1.06
Subjective WASO, min	22.7	34.9	18.8	44.9	0.09		−0.03 #		−0.06, 0.00 #
Objective WASO, min	76.1	56.8	86.2	64.9	0.62		3.66		−10.6, 17.9
Subjective TST, h	6.6	2.0	7.0	1.9	0.09		0.36		−0.05, 0.78
Objective TST, h	5.4	1.7	5.4	1.6	0.78		0.06		−0.36, 0.49

^a^ N = 27, ^b^ N = 29. Blood pressure, RHR = Resting heart rate, CRP = C-reactive protein, HDL = High-density lipoprotein, LDL = Low-density lipoprotein, HbA1c = Glycosylated hemoglobin. ^†^ *p*-values corrected using the false-discovery rate procedure. */** significant at *p* < 0.05/0.01 level. N.C.: *p*-values not corrected since variables were considered independent of cardiovascular outcomes. Mean, SD for post and pre are empirical values while the difference, *p*-values and 95% confidence intervals were based on either paired *t*-test or mixed models. The last six sleep variables in the table were measured at multiple occasions post and pre for the 30 shift workers and analyzed by linear mixed models. For all others, the difference between post and pre were analyzed using paired *t*-test. # Box–Cox transformed values.

## Data Availability

Not applicable.
